# Metabolic and transcriptomic analysis of two *Cucurbita moschata* germplasms throughout fruit development

**DOI:** 10.1186/s12864-020-6774-y

**Published:** 2020-05-15

**Authors:** Hafiz Muhammad Khalid Abbas, He-Xun Huang, An-Jun Wang, Ting-Quan Wu, Shu-Dan Xue, Aqeel Ahmad, Da-Sen Xie, Jun-Xing Li, Yu-Juan Zhong

**Affiliations:** Guangdong Key Laboratory for New Technology Research of Vegetables, Vegetable Research Institute, Guangdong Academy of Agricultural Sciences, Guangzhou, 510640 China

**Keywords:** *Cucurbita moschata*, Carotenoids, Sugars, Organic acids, Transcriptome

## Abstract

**Background:**

Pumpkins (*Cucurbita moschata*; Cucurbitaceae) are valued for their fruits and seeds and are rich in nutrients. Carotenoids and sugar contents, as main feature of pumpkin pulp, are used to determine the fruit quality.

**Results:**

Two pumpkin germplasms, CMO-X and CMO-E, were analyzed regarding the essential quality traits such as dry weight, soluble solids, organic acids, carotenoids and sugar contents. For the comparison of fruit development in these two germplasms, fruit transcriptome was analyzed at 5 different developmental stages from 0 d to 40 d in a time course manner. Putative pathways for carotenoids biosynthesis and sucrose metabolism were developed in *C. moschata* fruit and homologs were identified for each key gene involved in the pathways. Gene expression data was found consistent with the accumulation of metabolites across developmental stages and also between two germplasms. *PSY*, *PDS*, *ZEP*, *CRTISO* and *SUS*, *SPS*, *HK*, *FK* were found highly correlated with the accumulation of carotenoids and sucrose metabolites, respectively, at different growth stages of *C. moschata* as shown by whole transcriptomic analysis. The results of qRT-PCR analysis further confirmed the association of these genes.

**Conclusion:**

Developmental regulation of the genes associated with the metabolite accumulation can be considered as an important factor for the determination of *C. moschata* fruit quality. This research will facilitate the investigation of metabolic profiles in other cultivars.

## Background

The genus Cucurbita contains numerous species ranging from cultivated, *C. moschata* (*Cucurbita moschata* Duch.), *C. pepo* (*Cucurbita pepo* L.) and *C. maxima* (*Cucurbita maxima* Duch.) to several wild type species. Among these species, *C. moschata* is cultivated and consumed all over the world, and it provides good quality carotenoids and provitamin A. Moreover, there are abundant varieties for each pumpkin species which differ in shape, color and nutrient composition [1]. Pumpkin fruits and seeds are the rich source of nutrients including amino acids, flavonoids, phenolics and carbohydrates [2, 3]. Research has revealed its important medicinal aspects comprising antidiabetic, antioxidant, anticarcinomas and anticarcinogenic [4, 5]. Depending upon the environmental and storage conditions, mature fruits can be stored for minimum of 4 month or longer period. Pumpkin peels, which are discarded as agricultural by-products, contain about 10–40% of the carotenoids and provitamin A in total. The advantageous properties of carotenoids in pumpkin by-products have pronounced attraction for researchers and industrialists [6].

The main feature of pumpkin pulp is its carotenoids concentration, which gives flowers and fruits a coloration ranges from yellow to red. β-carotene (major carotenoid) and α-carotene are considered as the precursors of vitamin A, which is important for the normal growth of human body. Lutein could reduce the risk of certain eye disorders. Since they possess antioxidant activities, the consumption of carotenoids reduce the risk of several diseases including atherosclerosis, carcinomas and macular degeneration [7]. Several factors including maturation stage, growing environment and edaphoclimatic conditions can affect the composition of carotenoids in pumpkins. For example, studies have revealed the decreased biosynthesis of carotenoids in lower temperature areas [8, 9]. β-carotene and α-carotene are the main carotenoids in *C. moschata* varieties, while in many *C. maxima* varieties, lutein is detected as the main carotenoid [10]. Other important metabolites involved in the determination of pumpkin fruit quality are starch and sugar contents. Starch contents are considered important for fruit texture, e.g. the smoothness and dry consistency of squash is directly correlated with the high contents of starch and dry matter [11]. Similarly, adverse texture traits, fibrosity and wateriness are also associated with the low contents of starch and dry matter [11]. Sweetness of pumpkin fruit comes from the sugar contents, with sucrose as dominant [12]. Majorly, sweetness directly affect the consumer acceptability and overall flavor of squash [13]. Soluble solids, sucrose contents and sweetness are interconnected in pumpkin [14]. Hence, many of the important fruit quality aspects can be captured by measuring the carotenoids, starch and sugar contents.

Regardless of its economic importance, the genomes of *C. maxima* and *C. moschata* have been made accessible during recent years [15]. The availability of this genome is distinct from other cucurbits such as *C. lanatus*, *C. sativus* and *C. melo*, as the transcriptomes [16–18] and whole genome sequences [19–21] have already been reported. Until now, molecular level characterization of pumpkin (*C. moschata*) (2n = 2x = 40) was not seriously focused which delayed the developments for the consideration of its molecular biology and genetics. By the significant advancements in high-throughput sequencing techniques, generation of sequencing data for RNA-seq analysis has dramatically been increased recently [22], to provide the prompt and cost effective tools for the monitoring of transcriptomic variations. Numerous studies have reported the differential gene expression in different tissues at different growth and development stages under different environmental conditions [23]. In former studies, transcriptomic variations have been analyzed in response to single treatment, while the combination of different treatments has been extensively neglected. During the last few years, RNA-seq technology has been used intensively to investigate the transcriptomic variations within the species of Cucurbitaceae family, e.g. *C. lanatus* [16], *C. sativus* [17], *C. melo* [18], *Momordica cochnichinensis* [24], *Benicasa hispida* [25], *C. pepo* [26] and *C. maxima* [27].

The basic objectives of this study were to classify the unique transcriptional regulatory mechanisms in pumpkin (*C. moschata*) to recognize the important genes involved in the fruit quality formation and fruit ripening regulation. For this purpose, transcriptomic and metabolic analysis were performed on fruit pulp at different developmental stages. Finally, transcriptomic expression and metabolic profiles were comprehensively characterized and novel aspects of signaling pathways contributing in fruit development and ripening were uncovered. Findings from this study will help to design new strategies for the improvement of pumpkin molecular breeding.

## Results

### Increased contents of dry matter, brix and sugar

**Fig. 1:**
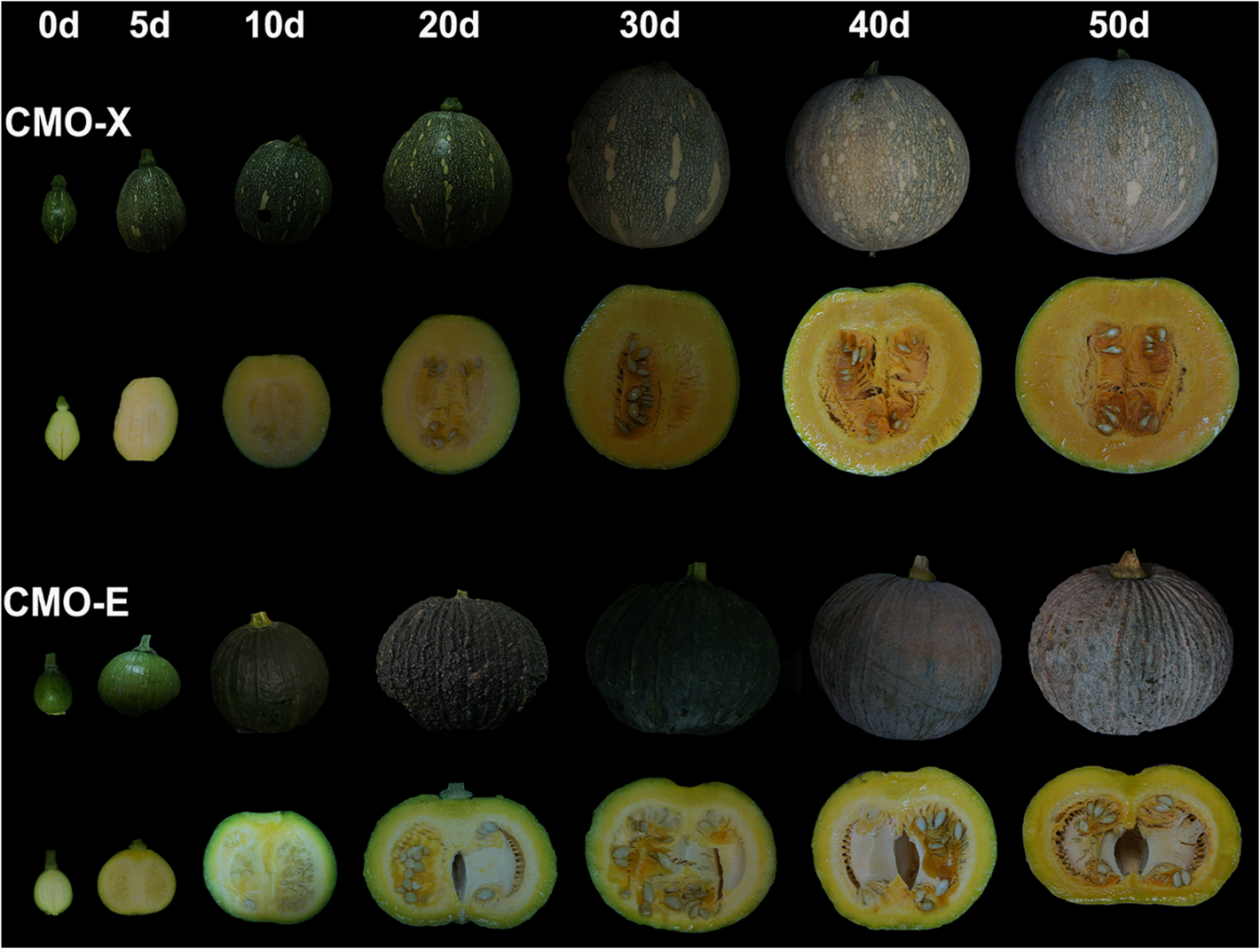
Different developmental stages of CMO-X and CMO-E fruit

For the determination of dry matter, brix, sugar, organic acids and carotenoids contents, fruit samples were collected and processed at different stages of development **(**Fig. 1**)**. Dry matter contents were recorded from 0 d to 50 d of fruit development, and found high from 10 d to 50 d of fruit development for CMO-X as compared to CMO-E. Similarly, the contents of total soluble solids (Brix) were high from 0 d to 40 d with gradual increase for CMO-X as compared to CMO-E. In case of CMO-X, the significant difference was observed between the brix values of 0 d (4.3) and 20 d (6.1) fruits, however, the brix values of 5 d and 10 d fruits were non significantly different than 0 d as well as 20 d. A non-significant difference was observed between the dry weight of 0 d (5.79) and 5 d (6.03) fruits of CMO-X **(**Table 1**)**.

**Table 1:** Dry weight and Brix in fruit tissues of CMO-X and CMO-E

		0d	5d	10d	20d	30d	40d	50d
**CMO-X**	DW (%)	5.79 ± 0.31^a^	6.03 ± 0.20^a^	12.06 ± 0.09^b^	14.38 ± 0.23^c^	16 ± 0.38^d^	19.27 ± 0.07^e^	21.08 ± 0.16^f^
	Brix	4.3 ± 0.14^a^	4.7 ± 0.36^ab^	5.4 ± 0.17^ab^	6.1 ± 0.22^b^	9.4 ± 0.39^c^	9.6 ± 0.15^c^	9.6 ± 0.06^c^
**CMO-E**	DW (%)	6.56 ± 0.18^a^	7.68 ± 0.20^b^	8.03 ± 0.27^b^	10.48 ± 0.23^c^	12.38 ± 0.32^d^	12.46 ± 0.08^d^	12.91 ± 0.12^d^
	Brix	4.2 ± 0.09^a^	4.2 ± 0.15^a^	4.5 ± 0.18^a^	6.6 ± 0.26^b^	7 ± 0.10^b^	8.6 ± 0.14^c^	11 ± 0.25^d^

Results are the averages from three individual experiments. ± indicate SD. Data were statistically analyzed by Duncan’s Multiple Range Test (DMRT) at *P* < 0.05 to determine significant differences among different time intervals mentioned by alphabetical letters (a, b, c…). *DW* Dry weight, Brix: Total soluble solids.

**Fig. 2:**
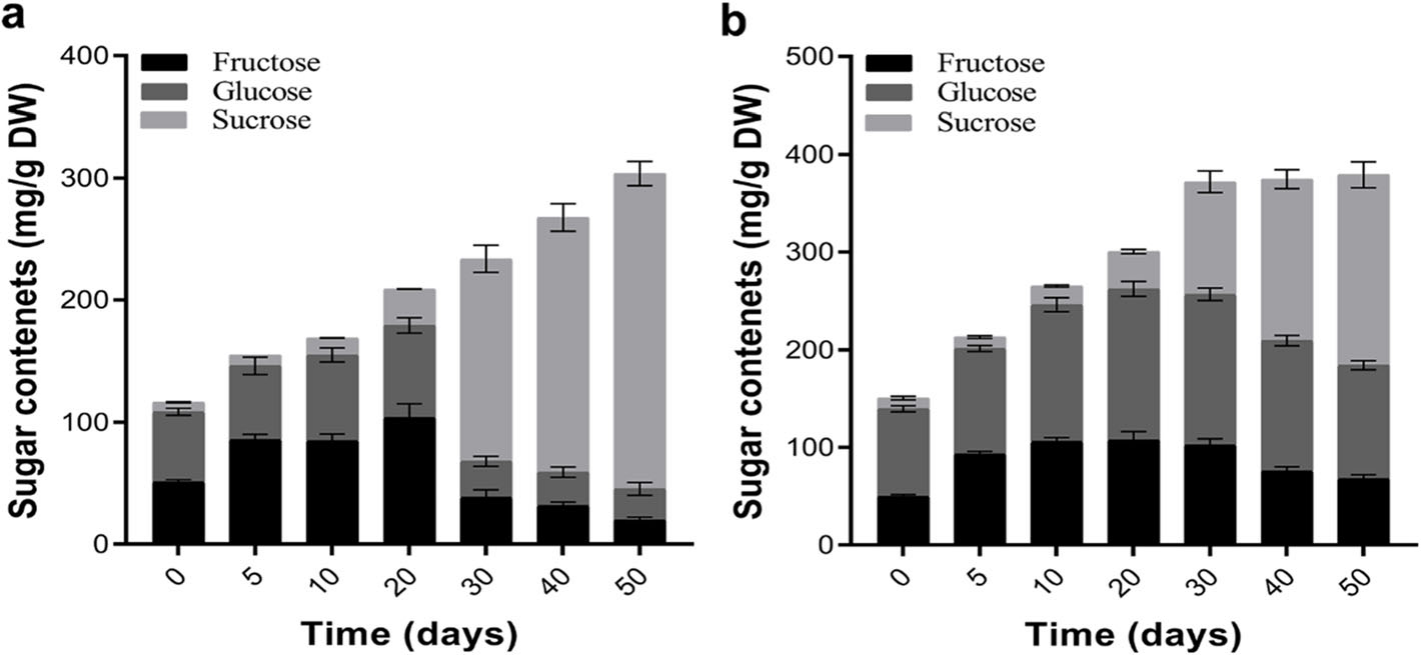
Sugar contents during different developmental stages of *C. moschata* fruit Samples were freeze dried and crushed into fine powder. HPLC connected to the refractive index (RI) detector was used for analysis of sugar contents. **a** Sugar contents in CMO-X, and (**b**) Sugar contents in CMO-E. Results are the averages from three individual experiments. Vertical bars represent SD.

Fructose, glucose and sucrose are the main sugars found in pumpkin fruit flesh. The contents of fructose and glucose were peaked at 20th day of fruit development, in case of CMO-X and CMO-E, and then started to decline. The high sucrose contents, 78.04 to 85.06% of the total sugars, during 30 d to 50 d of fruit development revealed CMO-X was significantly sweeter than CMO-E (Fig. 2a and b), as sucrose bears sweetness in pumpkins [12].

### Composition and contents of carotenoids and organic acids

**Fig. 3:**
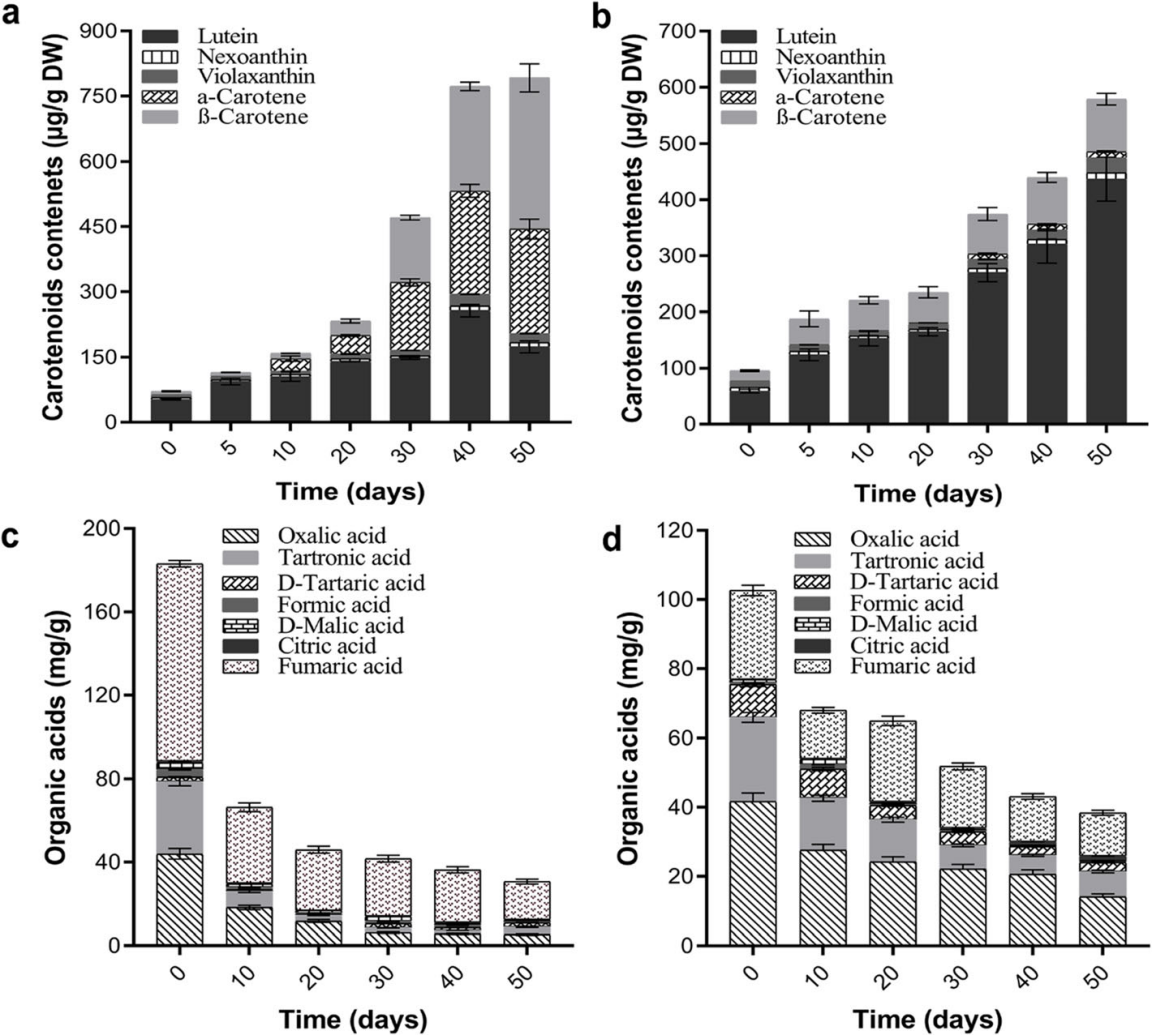
Carotenoids and organic acids during different developmental stages of *C. moschata* fruit. Samples were freeze dried and crushed into fine powder. Samples were analyzed using HPLC and then identification was performed by comparing the retention times and spectral data against known standards. **a** Contents of carotenoids in CMO-X, (**b**) Contents of carotenoids in CMO-E, (**c**) Different organic acids in CMO-X, and (**d**) Different organic acids in CMO-E. Data are the averages from three individual experiments. Vertical bars represent SD

Carotenoids are the key nutrients in pumpkin and offer orange color. α-carotene and β-carotene are the basic carotenoids among all others. Here, in this experiment, lutein, nexoanthin, vioaxanthin, α-carotene and β-carotenes were considered for analysis. In case of CMO-X, the maximum contents of lutein (256.61 μg/g DW) were recorded at 40 d of fruit development, while the contents of α-carotene (239.98 μg/g DW) and β-carotene (347.79 μg/g DW) were maximum at 50 d of fruit development (Fig. 3a). The contents of total carotenoids were maximum from 40 d to 50 d of fruit development in CMO-X. Similarly, in case of CMO-E, the contents of lutein (436.80 μg/g DW), α-carotene (11.03 μg/g DW) and β-carotene (92.71 μg/g DW) were increased gradually up to 50 d of fruit development (Fig. 3b). Due to the high accumulation of carotenoids (Fig. 3a), harvesting of CMO-X can be recommended during 40 d to 50 d of fruit development.

Organic acids are extensively distributed in different vegetables and fruits, and their quantity varies depending upon the biotic (species and cultivars) and abiotic factors (climate and soil). In *C. moschata*, different organic acids were analyzed at different fruit development stages. In case of CMO-X, the highest quantity of oxalic acid (43.89 mg/g), tartronic acid (34.71 mg/g), D-tartaric acid (2.28 mg/g), Formic acid (3.56 mg/g), D-malic acid (3.25 mg/g), citric acid (0.80 mg/g) and fumaric acid (94.62 mg/g) was present at 0 d of fruit development and then started to decline gradually (Fig. 3c). In case of CMO-E, maximum quantities of oxalic acid (41.68 mg/g), tartronic acid (24.17 mg/g), D-tartaric acid (9.58 mg/g) and fumaric acid (25.75 mg/g) were observed at 0 day of fruit development, while the quantities of formic acid (1.32 mg/g) and D-malic acid (1.52 mg/g) were highest at 10 d of fruit development. The contents of citric acid (1.38 mg/g) were gradually increasing up to 50 d of fruit development (Fig. 3d). These results indicated that, CMO-X and CMO-E must be harvested at early fruit development stages for the maximum utilization of organic acids.

### *C. moschata* transcriptome sequencing and unigene assembly

After removal of adaptor sequences and low-quality reads, a total of 65,126,810, 65,741,120, 50,804,292, 63,457,136 and 51,607,488 clean reads were obtained from 0 d, 10 d, 20 d, 30 d, and 40 d of *C. moschata* (CMO-X and CMO-E) fruit development (Additional file 1: Table S1). After de novo assembly of all 5 stages of *C. moschata*, 54.59 Mb transcriptome was obtained. A total of 55,158 unigenes were obtained with an average size and expression ratio of 989 bp and 99.53%, respectively, and these unigenes were aligned by 85.35% total reads and 72.89% unique reads (Additional file 1: Table S2). From the results of this assembly, 36,194 (65.61%), 30,244 (54.83%), 20,739 (37.59%) and 14,637 (26.53%) unigenes were annotated by Nr, Swiss-prot, KOG and KEGG databases, respectively (Additional file 1: Table S2). From Venn analysis, it was found that a total of 39,382 (71.39%) unigenes were annotated, among which 11,251 (28.56%) unigenes were annotated by four above mentioned databases (Additional file 1: Fig. S1a).

From the results of Nr alignment it has been observed that 12,709 (23.04%) and 10,502 (19.03%) unigenes showed highest homology to the genes from *Cucumis melo* and *C. sativus*, respectively. These results also revealed that *C. melo* and *C. sativus* are the most closely related species (Additional file 1: Figure S1b). All unigenes were classified into 25 KOG categories and 1542 (2.79%) unigenes were allocated to the carbohydrate transport and metabolism category (Additional file 1: Fig. S1c).

Gene ontology (GO) is a universal reliable gene functional classification system. To functionally characterize the DEGs in *C. moschata*, GO terms include the Biological processes, Molecular functions and Cellular components. A total of 14,637 unigenes were annotated against KEGG database and classified into 19, 12 and 17 functional groups, respectively (Additional file 1: Fig. S1d). In Biological processes category, 11,661 unigenes were allocated to the metabolic process which indicated that, *C. moschata* fruit flesh was going through extensive metabolic activities. In Molecular function category, 10,986 unigenes were allocated to the catalytic activity. While in Cellular component category, 9295 unigenes were assigned to the cell and cell part. It was also observed that, a total of 8132 members were assigned to the 125 different KEGG pathways (Additional file 1**: Table S3**). KEGG pathway enrichment analysis have shown that, 332 (4.08%) unigenes were allocated to the starch and sucrose metabolism, while 78 (0.96%) were allocated to the carotenoids biosynthesis (Additional file 1**: Table S3**).

### Gene expression analysis

**Fig. 4:**
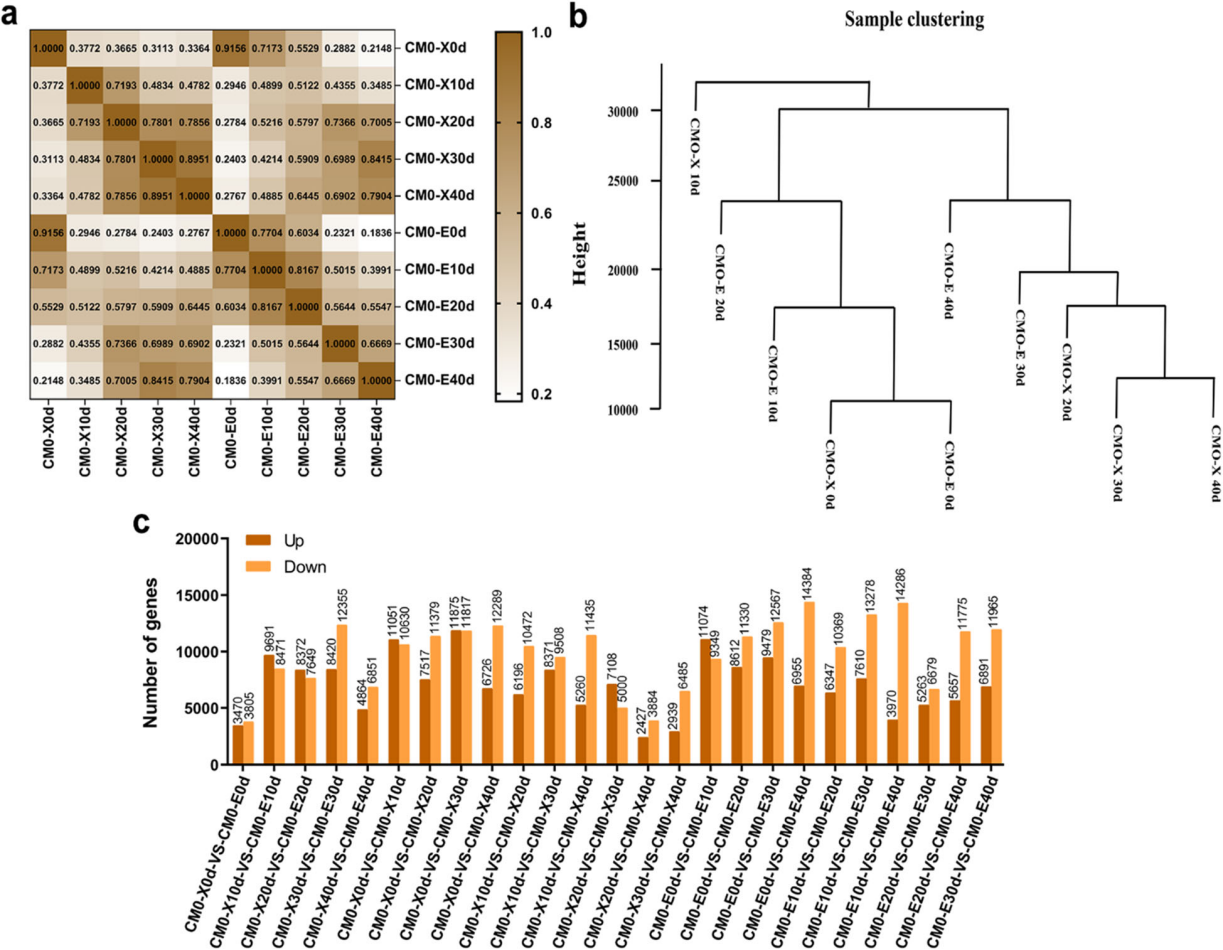
Pearson correlation, sample clustering and differentially expressed genes (DEGs) of *C. moschata*. Pearson correlation and sample clustering were performed to analyze the expression patterns of genes during different developmental stages of *C. moschata* fruit. **a** Pearson correlation, (**b**) Sample clustering, and (**c**) DEGs between CMO-X 0 d vs CMO-E 0 d, CMO-X 10 d vs CMO-E 10 d, CMO-X 20 d vs CMO-E 20 d, CMO-X 30 d vs CMO-E 30 d, CMO-X 40 d vs CMO-E 40 d, CMO-X 0 d vs CMO-X 10 d, CMO-X 0 d vs CMO-X 20 d, CMO-X 0 d vs CMO-X 30 d, CMO-X 0 d vs CMO-X 40 d, CMO-X 10 d vs CMO-X 20 d, CMO-X 10 d vs CMO-X 30 d, CMO-X 10 d vs CMO-X 40 d, CMO-X 20 d vs CMO-X 30 d, CMO-X 20 d vs CMO-X 40 d, CMO-X 30 d vs CMO-X 40 d, CMO-E 0 d vs CMO-E 10 d, CMO-E 0 d vs CMO-E 20 d, CMO-E 0 d vs CMO-E 30 d, CMO-E 0 d vs CMO-E 40 d, CMO-E 10 d vs CMO-E 20 d, CMO-E 10 d vs CMO-E 30 d, CMO-E 10 d vs CMO-E 40 d, CMO-E 20 d vs CMO-E 30 d, CMO-E 20 d vs CMO-E 40 d, CMO-E 30 d vs CMO-E 40 d

For the determination of gene expression, RPKM was considered as normalized expression value. RPKM values lower than 0.3 were filtered out and found that 82,761, 91,664, 83,007, 87,695 and 71,440 were total number of expressed genes retrieved from the reads of 0 d (CMO-X + CMO-E), 10 d (CMO-X + CMO-E), 20 d (CMO-X + CMO-E), 30 d (CMO-X + CMO-E) and 40 d (CMO-X + CMO-E) fruit flesh, respectively (Additional file 1**: Table S1**). Pearson correlation and sample clustering analysis were performed to investigate the unigene expression patterns in different developmental stages of *C. moschata* (CMO-X and CMO-E) fruit. Correlation of two parallel experiments provided the evaluation of the reliability of experimental results as well as operational stability. The correlation coefficient between two replicas was calculated to evaluate the repeatability between samples. The closer the correlation coefficient get to 1, the better the repeatability between two parallel experiments. CMO-X 0 d vs CMO-E 0 d showed the highest correlation coefficient than others (Fig. 4a). CMO-E 20 d data was similar to the CMO-X 10 d, CMO-E 10 d and CMO-E 40 d, while the data for CMO-E 30 d was similar with CMO-E 40 d and CMO-X 20 d. The data for CMO-X 0 d and CMO-E 0 d, and CMO-X 30 d and CMO-X 40 d were clustered together, respectively (Fig. 4b). This clustering analysis revealed that, gene expression pattern was similar for early stage of fruit development (CMO-X 0 d and CMO-E 0 d), and later stage of fruit development (CMO-X 30 d and CMO-X 40 d), while the gene expression pattern was found different for other developmental stages.

### Identification of differentially expressed genes (DEGs) and KEGG enrichment analysis

A threshold of |log_2_FC| ≥ 1 and FDR < 0.05 was used for the identification of DEGs in pairwise comparison. A total of 7275, 11,715, 19,015 and 21,339 unigenes were differentially expressed in CMO-X 0 d vs CMO-E 0 d, CMO-X 40 d vs CMO-E 40 d, CMO-X 0 d vs CMO-X 40 d and CMO-E 0 d vs CMO-E 40 d, respectively. Among these DEGs, 3470, 4864, 6726 and 6955 unigenes were upregulated, while 3805, 6851, 12,289 and 14,384 unigenes were downregulated in CMO-X 0 d vs CMO-E 0 d, CMO-X 40 d vs CMO-E 40 d, CMO-X 0 d vs CMO-X 40 d and CMO-E 0 d vs CMO-E 40 d pairwise comparison, respectively (Fig. 4c). DEGs have shown greater difference in 0 vs 10, 10 vs 20, 20 vs 30 and 30 vs 40 d analysis.

KEGG enrichment analysis of DEGs revealed the 86, 99 and 105 KEGG categories in total DEGs (Additional file 1**: Fig. S2a**), upregulated DEGs (Additional file 1**: Fig. S2b**) and downregulated DEGs (Additional file 1**: Fig. S2c**) from all pairwise comparisons, respectively. Starch and sucrose pathways were considerably enriched with total DEGs, upregulated and downregulated DEGs from CMO-X 20 d vs CMO-X 30 d, CMO-X 20 d vs CMO-X 30 d and CMO-X 10 d vs CMO-X 30 d, respectively (Additional file 1**: Fig. S2a-c**). Pentose and glucoronate interconversions were considerably enriched with total DEGs, upregulated and downregulated DEGs from CMO-E 20 d vs CMO-E 40 d, CMO-E 0 d vs CMO-E 30 d and CMO-X 20 d vs CMO-E 20 d, respectively (Additional file 1**: Fig. S2a-c**). It is concluded from this analysis that, activities related to the sugar accumulation in *C. moschata* fruit have already been started at early developmental stages. Carotenoids biosynthesis pathways were enriched with total DEGs, upregulated and downregulated DEGs from CMO-X 0 d vs CMO-X 20 d, CMO-X 30 d vs CMO-E 30 d and CMO-X 20 d vs CMO-X 30 d (Additional file 1**: Fig. S2a-c**).

### Genes involved in sucrose metabolism

**Fig. 5:**
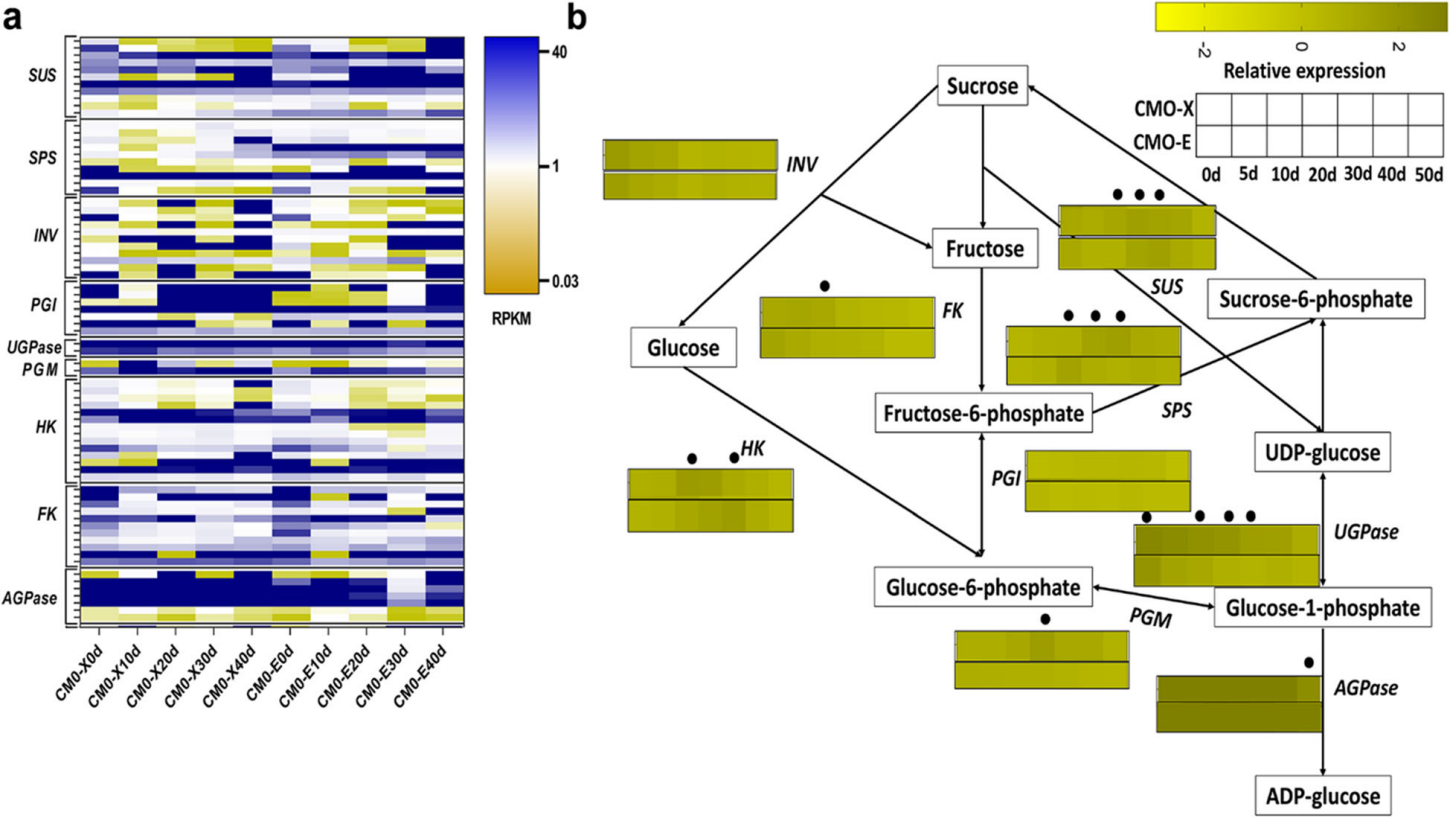
DEGs involved in sucrose metabolism of *C. moschata*. **a** Heat map showing the expression level (RPKM) of different unigenes from sucrose metabolism, (**b**) Proposed sucrose metabolism pathway in *C. moschata*, extracted from literature [28–32]. Gene expression (Relative expression) shown as heat maps, and time points with dots above them represented the significantly (*P* < 0.05) differentially expressed between two germplasms (CMO-X and CMO-E). Pathway genes and their abbreviations are as follow; *SUS* (Unigene0033931): Sucrose synthase, *SPS* (Unigene0040240): Sucrose phosphate synthase, *INV* (Unigene0044132): Sucrose invertase, *PGI* (Unigene0012171): Phosphoglucose isomerase, *UGPase* (Unigene0000830): UDP glucose pyrophosphorylase, *PGM* (Unigene0037284): Phosphoglucomutase, *HK* (Unigene0028620): Hexokinase, *FK* (Unigene0052295): Fructokinase and *AGPase* (Unigene0039030): ADP glucose pyrophosphorylase

Sucrose contents are the key components of pumpkin fruits. Here, a number of DEGs were observed to be involved in the pathways of sucrose metabolism. A total of 75 DEGs representing 9 genes were recognized on the basis of literature search, pathways and gene ontology, to be involved in sucrose metabolism (Fig. 5a, b and Additional file 1**: Table S4, Fig. S3**). These DEGs were assigned to 2 functional categories including sucrose synthesis (*SUS* and *SPS*) and sucrose degradation (*INV*, *PGI*, *UGPase*, *PGM*, *HK, AGPase*, and *FK*). *SUS* and *SPS* were expressing higher (RPKM> 12) from 0 d to 40 d for CMO-X and CMO-E fruit development. The homologs of *INV*, *HK* and *FK* were expressing lower in most of the fruit developmental stages for CMO-X and CMO-E. *AGPase* was expressing at high level (RPKM> 24) in all developmental stages for CMO-X and CMO-E. These results indicated that unigenes from sucrose pathways were expressing at different levels during expanding (20 d) and mature (40 d) stage of fruit development, to maintain the contents of sucrose in *C. moschata*.

### Genes involved in carotenoids biosynthesis pathways

**Fig. 6:**
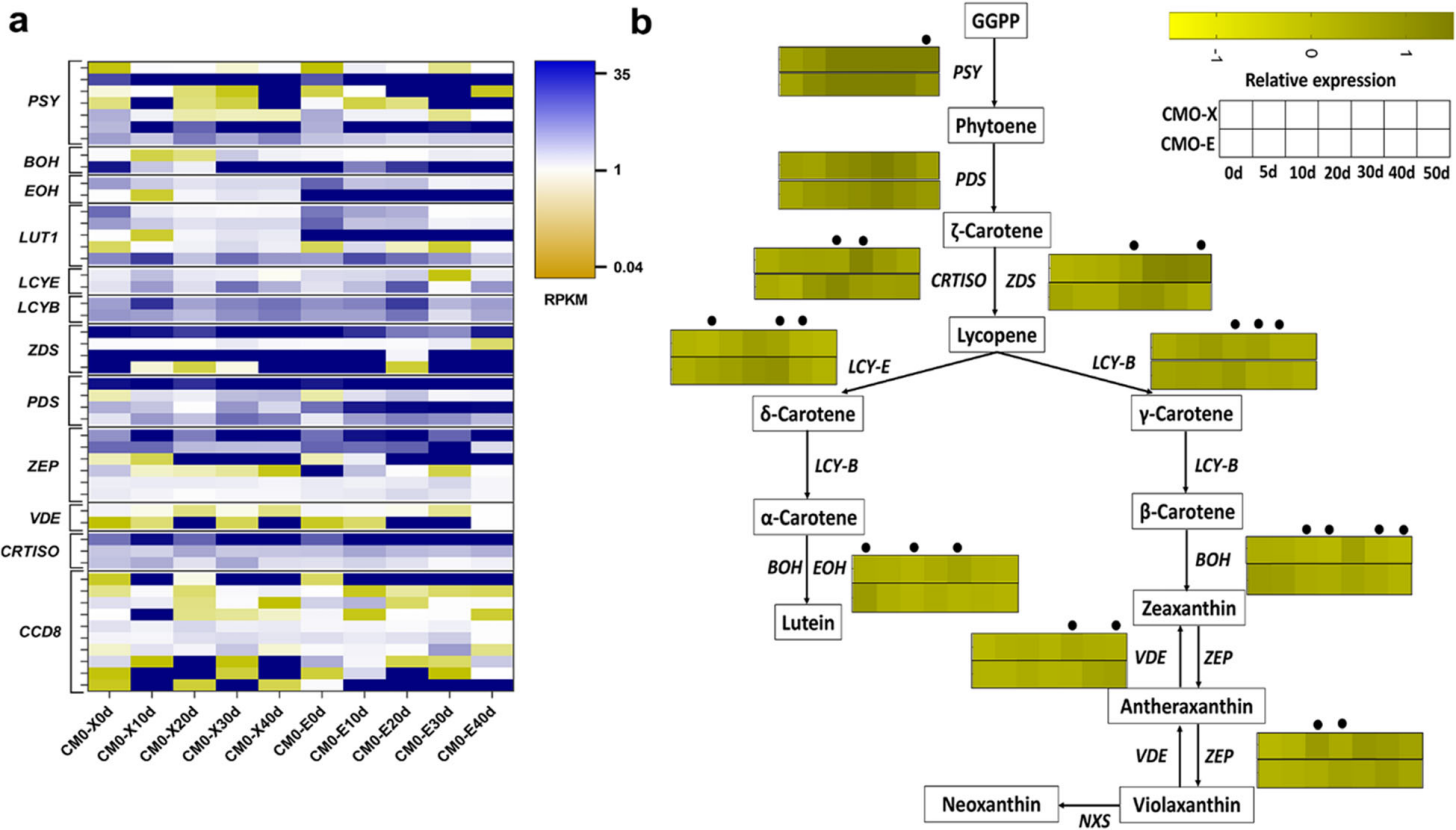
DEGs involved in carotenoids biosynthesis pathway of *C. moschata*. **a** Heat map showing the expression level (RPKM) of different unigenes from carotenoids biosynthesis, (**b**) Proposed carotenoids biosynthesis pathway in *C. moschata*, extracted from literature [33–36]. Gene expression (Relative expression) shown as heat maps, and time points with dots above them represented the significantly (*P* < 0.05) differentially expressed between two germplasms (CMO-X and CMO-E). Pathway genes and their abbreviations are as follow; *PSY* (Unigene0032135): Phytoene synthase, *BOH* (Unigene0031912): β-carotene hydroxylase, *EOH* (Unigene0042540): ɛ-carotene hydroxylase, *LUT1*: β-ring hydroxylase, *LCY-E* (Unigene0031052): Lycopene-ɛ-cyclase, *LCY-B* (Unigene0018220): Lycopene-β-cyclase, *ZDS* (Unigene0035295): ζ-carotene, *PDS* (Unigene0036921): Phytoene desaturase, *ZEP* (Unigene0003183): Zeaxanthin epoxidase, *VDE* (Unigene0053140): Violaxanthin de-epoxidase, *CRTISO* (Unigene0036596): Carotenoid isomerase and *CCD8*: Carotenoid cleavage dioxygenase

Carotenoids concentration is the main feature which gives an esthetic and nutritional value to pumpkin fruit. Forty-nine DEGs representing 12 genes were recognized on the basis of literature search, pathways and gene ontology, to be involved in carotenoids biosynthesis in *C. moschata* (Fig. 6a, b and Additional file 1**: Table S5, Fig. S4**). Four different genes, *PSY*, *ZDS, PDS* and *CRTISO*, involved in carotenoids synthesis were expressing higher (RPKM> 18) in all fruit developmental stages of CMO-X and CMO-E. The expression level of *PSY* was higher from 10 d to 40 d of fruit development for CMO-X CMO-E, while the expression level of *ZDS* was higher from 0 d to 40 d for CMO-X and 0 d to 10 d and 30 d to 40 d for CMO-E fruit development. The expression level of *PDS* was high from 0 d to 40 d for CMO-X and CMO-E. The expression of *CRTISO* was higher at 10 d and 30 d to 40 d for CMO-X, and 10 d to 40 d for CMO-E. *LCYE*, lutein synthesis gene, was expressing high (RPKM> 5) from 10 d to 30 d for CMO-X and 5 d to 40 d for CMO-E fruit development. *BOH* and *VDE* represents the zeaxanthin synthesis in pumpkin. Expression of *BOH* was higher at 0 d and 30 d to 40 d for CMO-X, and 0 d to 40 d for CMO-E fruit development, while the expression of *VDE* was higher at 20 d and 40 d for CMO-X, and 20 d to 30 d CMO-E fruit development. The expression of *EOH* was lower in CMO-X, while in CMO-E, it was expressing higher during all fruit development stages to verify the higher contents of lutein. Other genes, *LUT1*, *LCYB*, *ZEP* and CCD8 were also expressing at different levels in different fruit development stages.

### Verified relative expression of different genes from carotenoids and sucrose biosynthesis pathways

To verify the expression of the different DEGs from sucrose, and carotenoids biosynthesis pathways, 19 different genes were confirmed by qRT-PCR using gene specific primers. These selected genes were *PSY*, *PDS*, *ZDS*, *LCYE*, *LCYB*, *EOH*, *BOH*, *VDE*, *ZEP* and *CRTISO* from carotenoids biosynthesis pathways, and *INV*, *SUS*, *SPS*, *HK*, *FK*, *PGI*, *UGPase*, *PGM* and *AGPase* from sucrose pathways. From the results, it was observed that the expression patterns of nominated genes produced by qRT-PCR were similar with the expression patterns obtained from RNA-seq data (Additional file 1**: Fig. S3a-d and S4a-d**). Comparative gene expressions were determined by 2^∆∆Ct^ method.

## Discussion

Pumpkin is famous for the accumulation of sugar and carotenoids among all known Cucurbitaceae plants. For the development of high-quality pumpkin fruit, fruit ripening process undergoes several different stages to accumulate the nutrients, physiological and sensory chemicals. In this study, metabolic and transcriptomic analyses of two germplasms (CMO-X and CMO-E) of *C. moschata* were used to explore the fruit ripening and quality at different developmental stages.

### Composition and contents of dry matter, brix, carotenoids, organic acids and sugar

Fruit quality is one of the imperative factor for the pumpkin breeding. Carotenoids, sugars and organic acids are considered to be the main metabolites involved in the determination of fruit quality. Among them, starch and sugar contents give special texture and sweetness to the fruit, respectively. Total soluble solids are correlated with sucrose contents and perceived sweetness of the fruit [11, 12, 14]. It is possible to capture the several fruit quality aspects by measuring the sugar contents. Initially, dry weight and brix were measured in all developmental stages of fruit from 0 to 50 d, and a continuous and gradual increase was observed. In sugar composition analysis of *C. moschata*, the contents of sucrose were continuously increasing during all developmental stages and declared as dominant sugar in *C. moschata*. These results are also supported by the previous literature [14, 27, 37] to conclude the fact that, the phloem translocation and rapid fruit growth can be considered as major factors for this phenomenon.

Carotenoids are also the important factor which determine the pumpkin fruit quality, as it provides fruit color and nutritional benefits [38]. These carotenoids are differently distributed among different pumpkin species, e.g. β-carotene, lutein and violaxanthin are the main carotenoids in *C. maxima*, while in *C. pepo*, lutein and β-carotene behave as principal carotenoids. In *C. moschata*, α-carotene and β-carotene are detected as main carotenoids [10]. Surprisingly, in CMO-X germplasm, α-carotene, β-carotene and lutein were detected as primary carotenoids, while β-carotene and lutein were detected as primary carotenoids in CMO-E germplasm. The interesting composition of carotenoids in CMO-X and CMO-E can be explained with the fact that these two germplasms were generated from the hybridization of *C. maxima* and *C. moschata*. Our results indicated the consistency with the study where *C. maxima* and *C. moschata* were hybridized to produce a cultivar “Maxchata” which contains violaxanthin, lutein and β-carotene as principal carotenoids [33]. From present study, it is concluded that different hybridization patterns among *C. maxima*, *C. pepo* and *C. moschata* can be designed to produce different pumpkin cultivars with desired pattern of carotenoids.

In vegetables, organic acids are present in low concentrations and play their role to impart the antimicrobial activities and to enhance the flavor. To indicate the ripening of fruits and vegetables, proportion of organic acid to that of sugars is used as scale [39]. Organic acids especially citric and malic acids play their role to inhibit the growth of undesired microorganisms in human body and also influence the metabolic process. Additionally, organic acids possess antioxidant potential and exert their protective role against variety of pathogens [40, 41]. In our study, significant contents of citric and malic acids were detected along with several other organic acids. The accumulation pattern of these organic acids was different for each individually detected organic acid. On the basis of these findings, CMO-X and CMO-E can be recommended for the investigation of antimicrobial genes which can be used in the field of drug discovery.

### *C. moschata* fruit transcriptome assembly

Transcriptomic analysis has become viable and active approach to investigate the gene expression profiles in different crops and identify candidate genes [42]. As no previous reports are available regarding to the *C. moschata* fruit transcriptomic analysis, so in present study, we have sequenced and assembled transcriptomic data from *C. moschata* fruit development. Our fruit transcriptome covers data from two germplasms (CMO-X and CMO-E) of *C. moschata* with an average length of 989 bp. This transcriptome has greater number of unigenes (55,158) than the published transcriptome of *C. maxima* fruit [27] and lesser than the *C. pepo* fruit [38]. On the basis of homologs identification of all metabolic genes of interest, the transcriptome has successfully captured gene expression during all developmental stages. In our results, a high percentage (11,661) of unigenes were involved in metabolic process which indicated that molecular regulation of fruit ripening was an active and complex physiological event. Additionally, carotenoids biosynthesis, sucrose metabolism, and pentose and glucoronate interconversions were enriched in KEGG pathways enrichment analysis. These pathways have also been reported for fruit ripening in orange [43], melon [44] and watermelon [45]. These pathways are also considered to play their role for fruit development regulation in climacteric and non-climacteric plants [45, 46].

### Sucrose metabolism pathway

For metabolites biosynthesis and other developmental events, sugars are transported to the fruit in early developmental stages. Among number of different sugars, fructose, glucose, sucrose and galactose are also transported [47]. Sucrose can be broken down either by sucrose invertase (*INV*) or sucrose synthase (*SUS*). In our experiment, higher expression of invertase was observed at early fruit development stages, while during later stages of fruit development, the expression of sucrose synthase was higher. These results are consistent with previous study in buttercup squash [48] where invertase activity was observed high at the start of fruit development. These results suggested the high osmotic potential for rapid fruit expansion at early stages. *UGPase* and *PGM* were expressing at higher level throughout all developmental stages to justify their part in synthesizing glucose-6-phosphate which is used as substrate for starch synthesis in amyloplast. In another step, *PGM* acts on glucose-6-phosphate to synthesize glucose-1-phosphate, and then *AGPase* (important in determining the level of starch accumulation) converts it into ADP-glucose for starch (amylose and amylopectin) synthesis [37, 49, 50]. Several enzymes i.e. *GBSS* converts ADP-glucose into amylose, an unbranched form of starch, and *SS* and *SBE* synthesize amylopectin, branched form of starch [37].

### Carotenoids biosynthesis pathway

Carotenoids are not only important to the organisms where they synthesized but also to the other organisms, as they have been recognized as important nutrients and health beneficial elements [34]. Phytoene synthase (*PSY*) was involved in the conversion of geranylgeranyl pyrophosphate (GGPP) into phytoene [51]. *PSY* has also been reported as key enzyme to limit the carotenoid concentration in watermelon [16] . In our study, higher expression of *PSY* was observed during 10 d to 50 d of CMO-X fruit development, which is positively correlated with observed total carotenoids contents. The high expression of *PSY* also indicated its central role in the regulation of carotenoids biosynthesis pathway. *LCY-B* and *LCY-E* convert lycopene into α-carotene and then lutein, or *LCY-B* exclusively can act to make β-carotene. Natural variations in *LCY-E* can affect the type of carotenoids by two branches of the pathway [52]. In our experiment, we observed the high expression of *ZEP* during 30 d to 50 d of CMO-X and 20 d to 50 d of CMO-E fruit development, which is directly correlated with accumulation of violaxanthin in fruit tissue. The expression of *EOH* was high from 20 d to 30 d for CMO-X and at 0 d and 20 d for CMO-E, while the expression of *BOH* was higher from 0 d to 5 d and 30 d for CMO-X and CMO-E fruit development, although the contents of lutein in our *C. moschata* were significantly high through all developmental stages. The other genes i.e. *PDS*, *CRTISO* and *VDE* were expressing at different levels during different fruit development stages to justify their role in carotenoids biosynthesis pathway.

## Conclusion

In this study, fruit transcriptome of *C. moschata* (CMO-X and CMO-E) was sequenced across different developmental stages for the 1st time to explore the expression of key genes involved in the metabolic processes that affect the fruit quality. These two germplasms showed differences in essential fruit quality traits of percent dry weight, soluble solids, organic acids, carotenoids and sugar contents. Putative pathways for carotenoids biosynthesis and sucrose metabolism were assembled from previous literature and expression of key genes varied at different fruit developmental stages and between two germplasms. The gene expression data was consistent with the accumulation of metabolites in fruit developmental stages of *C. moschata* germplasms. On the basis of these results, future research can be designed to determine the expression patterns of these key genes in different cultivars to investigate the metabolic profiles responsible for the fruit quality. These findings will also help to design new strategies for the improvement of pumpkin molecular breeding.

## Methods

### Pumpkin fruit sampling

The germplasms of *C. moschata* (CMO-X and CMO-E) were grown under controlled conditions of 18 °C–26 °C and 15 h/9 h of light/dark intervals in greenhouses of the Guangdong Academy of Agricultural Sciences (GDAAS), Guangzhou, P. R. China. Fruit samples were collected at specific time points (0, 5, 10, 20, 30, 40 and 50 d) after pollination. Minimum of 3–4 fruits were collected at every stage for analysis. Fruit pulp was collected and immediately transferred to the liquid nitrogen and finally preserved at − 80 °C for future analyses.

### Measurement of dry matter, brix, carotenoids, organic acids and sugar contents

For the measurement of metabolites, fruit pulp was homogenized in blender (Philips HR7628/00). For the measurement of dry matter, samples were desiccated at 65 °C for 36 h to achieve the persistent weight. Percentage of final weight comparative to the initial weight was considered to determine the dry matter. ATAGO PAL-2 refractometer was used for the measurement of Brix. Minimum of three replicates were used for each measurement.

For the analysis of carotenoids and sugar contents, freeze dried samples were used. Samples were crushed into fine powder and preserved at − 80 °C. HPLC was used to detect the carotenoid contents according to the protocol described previously [53]. Evaluation was carried out by the integration of peak areas and then changed them into the concentrations by comparing with purchased standards (Sigma). Describing in details, an isotopically labelled internal standard was used for each target compound**.** Separate calibration curves (CCv) were prepared and their response factors were calculated using the formula R = Conc_c_ /A_c_. While R was the response factor; Conc_c_ was the known concentration of the standard compound at a fixed point of interception in the CCv, and A_c_ was the value of peak area at the interception point. The target compound was quantified by using the formula Conc_t_ = R × A_t_. While Conc_t_ was the concentration of the target compound and A_t_ was the experimental peak area of the target compound. A cocktail of the internal standard compounds was prepared with the fixed quantity of 1.0 μg/ mL and subjected to a chromatographic run to check the peak separation efficiency and closely similar retention time of the target compounds. The cocktail was added into the sample (maintaining the fixed concentration of 1.0 μg/ mL of each standard compound) for the compensation of variability sources and tracking the target compounds. Then the peak area of a target compound was pointed out at the respective CCv, and used to determine the specific compound concentration residing at the perpendicular axis. Sugar contents were detected by using HPLC system coupled with Refractive index (RI) detector with minor modifications in previously described protocol [54]. In the modified method, the loaded samples were separated at 40 °C by using Xbridge Amide (Waters, 4.6 mm, × 150 mm, 3.5 μm). Acetonitrile/deionized water, 8:2 (v/v), was used at mobile phase at flow rate of 1 ml/min. Quantification of sugars was performed by comparing with purchased standards (Sigma). Organic acids were measured according to the previous protocol [55].

### Construction of *C. moschata* cDNA library

Fruit pulp was collected at five different stages (0, 10, 20, 30 and 40 d) and saved at − 80 °C. At each stage, three replicates were collected and pulp was mixed to homogenize. Trizol reagent was used to extract the total RNA, which was then purified with DNaseI. Agarose gel (2%) was used to check the integrity of purified RNA and then it was quantified on ND-2000 spectrophotometer. NEBNext® Ultra™ II RNA Library Prep Kit from Illumina® was used to construct the libraries according to manufacturer’s instructions. Following the PCR amplification, Agilent 2100 Bioanalyzer (Agilent, San Diego, CA, USA) was used to validate the libraries. Finally, sequencing was performed at Gene Denove Biotechnology Co. (GuangZhou, China) on Illumina HiSeq™ 4000 sequencing platform (Illumina, San Diego, CA, USA).

### Raw data processing and functional annotation

To get high quality clean reads, Fastp [56] (version 0.18.0) was used with following parameters: 1) removal of reads with adapters; 2) removal of reads with > 10% of unknown nucleotides (N) and 3) removal of low quality reads with > 50% of low quality (Q value≤20) bases. De novo transcriptome assembly was carried out with Trinity [57], a short reads assembling program and then aligned with @blastdb using BLASTx (e-value < 0.00001). Different databases (Nr, Swiss-Prot, KEGG and COG) were selected to confirm the sequence directions. Finally, alignment was carried out via BLASTx between unigenes and protein databases of Nr, Swiss-Prot, KEGG and COG, and retrieved protein sequences with highest similarity were used for functional annotation and classification. Blast2GO program [58] was used for Gene Ontology (GO) functional annotation. For GO functional classification of all unigenes, WEGO software was used according to description [59]. The raw data is available on NCBI database under accession: PRJNA629657.

### Unigene differential expression analysis

Bowtie [60] was used for the alignment of clean reads to assemble the transcripts. Reads per kilobase per million mapped reads (RPKM), calculated by RSEM (RNA-Seq by Expectation Maximization) [61], were considered to express the quantity of gene expression, thus neglecting the impact of sequencing length and differences. For differentially expressed genes (DEGs) significance analysis, a threshold of FDR (False Discovery Rate) < 0.05, |log_2_FC| ≥ 1 was used. Nr database and KEGG pathway database were used for the annotation of all genes. The KEGG enrichment analysis was achieved with Q-value cut-off of 0.05. Sample clustering analysis and Pearson correlation were performed using “fastcluster” and “cor” function in R 3.5.1 (http://www.r-project.org/), respectively.

### Relative gene expression verification by qRT-PCR

From pumpkin fruits, total RNA was extracted and cDNA was synthesized using Superscript reverse transcriptase (Invitrogen, Cartsbad, CA, USA) with oligo dT primers. Concentrations of cDNA were achieved to be equal. qRT-PCR analysis was carried out to analyze the relative expression level of genes from sucrose and carotenoids biosynthetic pathways, using SYBR® Premix Ex TaqTMII (TliRNaseH Plus) (TaKaRa Clontech). The gene specific primers presented in Additional file 1**: Table S6**. Actin was used as reference gene. BIO-RAD Cycler IQ Multi-Color Real-Time PCR Detection System (BIO-RAD, Hercules, CA, USA) was used to process the PCR mixture under following program: 95 °C for 30 s followed by 40 cycles of 95 °C for 5 s and 60 °C for 30 s. A melting curve was adjusted from 65 °C to 95 °C. Three replications were selected for each fruit from each stage and also for control. The 2^-ΔΔCT^ method [62] was used for the quantification of relative expression.

## Supplementary information

**Additional file 1: Table S1.** No of genes in different *C. moschata* fruit samples. **Table S2.** Statistic of sequencing and de novo assembling of transcriptome in *C. moschata*. **Figure S1.** Annotation of *C. moschata* unigenes. **Table S3**. No of unigenes assigned to different types of *C. moschata* pathways. **Figure S2**. KEGG pathways enrichment analysis of DEGs of *C. moschata*. **Table S4**. Genes involved in sucrose metabolism. **Figure S3**. Relative expression and RPKM of different genes from sucrose pathway. **Table S5**. Genes involved in carotenoids biosynthesis pathways. **Figure S4**. Relative expression and RPKM of different genes from carotenoids biosynthesis pathway. **Table S6**. List of primers used for qRT-PCR.

## Data Availability

The data sets generated and analyzed during the current study are available on NCBI database under accession: PRJNA629657/https://www.ncbi.nlm.nih.gov/bioproject/629657

## Abbreviations

SUS: Sucrose synthase
SPS: Sucrose phosphate synthase
INV: Sucrose invertase
PGI: Phosphoglucose isomerase
UGPase: UDP glucose pyrophosphorylase
PGM: Phosphoglucomutase
HK: Hexokinase
FK: Fructokinase
AGPase: ADP glucose pyrophosphorylase
PSY: Phytoene synthase
BOH: β-carotene hydroxylase
EOH: ɛ-carotene hydroxylase
LUT1: β-ring hydroxylase
LCY-E: Lycopene-ɛ-cyclase
LCY-B: Lycopene-β-cyclase
ZDS: ζ-carotene
PDS: Phytoene desaturase
ZEP: Zeaxanthin epoxidase
VDE: Violaxanthin de-epoxidase
CRTISO: Carotenoid isomerase
CCD8: Carotenoid cleavage dioxygenase
