# Supplementary material for: Metabolic and transcriptomic analysis of two Cucurbita moschata germplasms throughout fruit development
Source: BMC Genomics. 2020 May 15;21:365. doi: 10.1186/s12864-020-6774-y (PMC7227267; doi:10.1186/s12864-020-6774-y)
Supplement: Supplementary file 1 — Additional file 1: Table S1. No of genes in different C. moschata fruit samples. Table S2. Statistic of sequencing and de novo assembling of transcriptome in C. moschata. Figure S1. Annotation of C. moschata unigenes. Table S3. No of unigenes assigned to different types of C. moschata pathways. Figure S2. KEGG pathways enrichment analysis of DEGs of C. moschata. Table S4. Genes involved in sucrose metabolism. Figure S3. Relative expression and RPKM of different genes from sucrose pathway. Table S5. Genes involved in carotenoids biosynthesis pathways. Figure S4. Relative expression and RPKM of different genes from carotenoids biosynthesis pathway. Table S6. List of primers used for qRT-PCR. [file 12864_2020_6774_MOESM1_ESM.docx]

**Supplementary**

**Metabolic and transcriptomic analysis of two *Cucurbita moschata* germplasms throughout fruit development**

Hafiz Muhammad Khalid Abbas^†^, He-Xun Huang^†^, An-Jun Wang, Ting-Quan Wu, Shu-Dan Xue, Aqeel Ahmad, Da-Sen Xie, Jun-Xing Li, Yu-Juan Zhong^*^

**Table S1** No of genes in different *C. moschata* fruit samples

| **Sample** | **Clean reads** | **No. of expressed genes (ratio)** | **New genes** | **All gene** |
| --- | --- | --- | --- | --- |
| CMO-X0d | 33,933,616 | 41,796 (75.78%) | 0 | 41,796 |
| CMO-X10d | 31,440,236 | 45,607 (82.69%) | 0 | 45,607 |
| CMO-X20d | 21,826,588 | 39,412 (71.46%) | 0 | 39,412 |
| CMO-X30d | 34,275,828 | 45,139 (81.84%) | 0 | 45,139 |
| CMO-X40d | 21,796,970 | 36,675 (66.50%) | 0 | 36,675 |
| CMO-E0d | 31,193,194 | 40,965 (74.27%) | 0 | 40,965 |
| CMO-E10d | 34,300,884 | 46,057 (83.51%) | 0 | 46,057 |
| CMO-E20d | 28,977,704 | 43,595 (79.04%) | 0 | 43,595 |
| CMO-E30d | 29,181,308 | 42,556 (77.16%) | 0 | 42,556 |
| CMO-E40d | 29,810,518 | 34,765 (63.03%) | 0 | 34,765 |

**Table S2** Statistic of sequencing and *de novo* assembling of transcriptome in *C. moschata*

|  | **No of unigenes** | **No of bp** | **No of reads** |
| --- | --- | --- | --- |
| **Total unigenes** | 55,158 |  |  |
| Nr annotation | 36,194 |  |  |
| Swiss-Prot annotation | 30,244 |  |  |
| KOG annotation | 20,739 |  |  |
| KEGG annotation | 14,637 |  |  |
| **Total annotated genes** | 39,382 |  |  |
| Without annotation | 1576 |  |  |
| Max. length |  | 14341 |  |
| Mini. Length |  | 201 |  |
| Mean length |  | 989 |  |
| Total size |  | 54,595,577 |  |
| **Total reads** |  |  | 296,736,846 |
| Mapped reads |  |  | 253,259,057 |
| Unique mapped reads |  |  | 216,292,854 |
| **RPKM=0** | 258 |  |  |


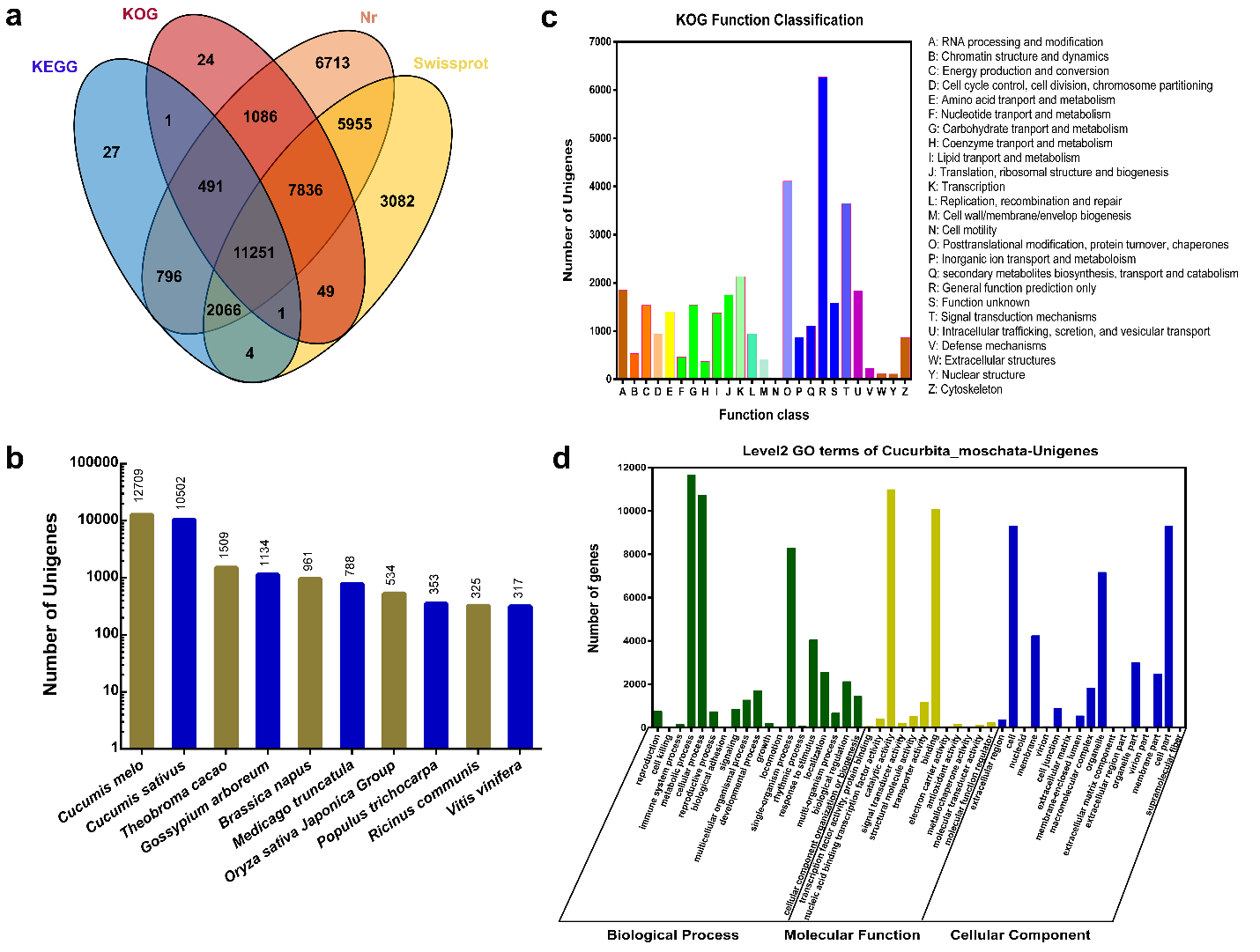


**Fig. S1** Annotation of *C. moschata* unigenes. **(a)** Venn diagram showing the annotation of unigenes from four different (KEGG, KOG, Nr and Swiss-Prot) databases, **(b)** KOG function classification of all unigenes, **(c)** Species distribution of Nr (non-redundant) unigene annotation and **(d)** Distribution of GO annotation of all unigenes.

**Table S3** No of unigenes assigned to different types of *C. moschata* pathways

| **Sr. No** | ***C. moschata* pathways** | **No of unigenes with pathway annotation (8132)** | **Pathway IDs** |
| --- | --- | --- | --- |
| **1** | Carbon metabolism | 608 (7.48%) | ko01200 |
| **2** | Ribosome | 571 (7.02%) | ko03010 |
| **3** | Biosynthesis of amino acids | 547 (6.73%) | ko01230 |
| **4** | Plant hormone signal transduction | 441 (5.42%) | ko04075 |
| **5** | Purine metabolism | 335 (4.12%) | ko00230 |
| **6** | Starch and sucrose metabolism | 332 (4.08%) | ko00500 |
| **7** | Protein processing in endoplasmic reticulum | 297 (3.65%) | ko04141 |
| **8** | Spliceosome | 296 (3.64%) | ko03040 |
| **9** | Oxidative phosphorylation | 284 (3.49%) | ko00190 |
| **10** | Endocytosis | 280 (3.44%) | ko04144 |
| **11** | Pyrimidine metabolism | 261 (3.21%) | ko00240 |
| **12** | RNA transport | 261 (3.21%) | ko03013 |
| **13** | Plant-pathogen interaction | 234 (2.88%) | ko04626 |
| **14** | Glycolysis / Gluconeogenesis | 230 (2.83%) | ko00010 |
| **15** | Amino sugar and nucleotide sugar metabolism | 227 (2.79%) | ko00520 |
| **16** | Pyruvate metabolism | 218 (2.68%) | ko00620 |
| **17** | Ubiquitin mediated proteolysis | 211 (2.59%) | ko04120 |
| **18** | Cysteine and methionine metabolism | 194 (2.39%) | ko00270 |
| **19** | Phenylpropanoid biosynthesis | 193 (2.37%) | ko00940 |
| **20** | mRNA surveillance pathway | 177 (2.18%) | ko03015 |
| **21** | Carbon fixation in photosynthetic organisms | 177 (2.18%) | ko00710 |
| **22** | RNA degradation | 173 (2.13%) | ko03018 |
| **23** | Glyoxylate and dicarboxylate metabolism | 169 (2.08%) | ko00630 |
| **24** | Glycine, serine and threonine metabolism | 165 (2.03%) | ko00260 |
| **25** | Alanine, aspartate and glutamate metabolism | 162 (1.99%) | ko00250 |
| **26** | Glycerophospholipid metabolism | 159 (1.96%) | ko00564 |
| **27** | Peroxisome | 155 (1.91%) | ko04146 |
| **28** | Photosynthesis | 152 (1.87%) | ko00195 |
| **29** | Glutathione metabolism | 145 (1.78%) | ko00480 |
| **30** | Fatty acid metabolism | 145 (1.78%) | ko01212 |
| **31** | 2-Oxocarboxylic acid metabolism | 137 (1.68%) | ko01210 |
| **32** | Aminoacyl-tRNA biosynthesis | 129 (1.59%) | ko00970 |
| **33** | Pentose and glucuronate interconversions | 124 (1.52%) | ko00040 |
| **34** | Fructose and mannose metabolism | 123 (1.51%) | ko00051 |
| **35** | Citrate cycle (TCA cycle) | 122 (1.5%) | ko00020 |
| **36** | Ribosome biogenesis in eukaryotes | 121 (1.49%) | ko03008 |
| **37** | Valine, leucine and isoleucine degradation | 119 (1.46%) | ko00280 |
| **38** | Phagosome | 118 (1.45%) | ko04145 |
| **39** | Pentose phosphate pathway | 118 (1.45%) | ko00030 |
| **40** | Galactose metabolism | 116 (1.43%) | ko00052 |
| **41** | Arginine and proline metabolism | 113 (1.39%) | ko00330 |
| **42** | Phosphatidylinositol signaling system | 111 (1.36%) | ko04070 |
| **43** | Inositol phosphate metabolism | 110 (1.35%) | ko00562 |
| **44** | Terpenoid backbone biosynthesis | 108 (1.33%) | ko00900 |
| **45** | Phenylalanine, tyrosine and tryptophan biosynthesis | 104 (1.28%) | ko00400 |
| **46** | Proteasome | 102 (1.25%) | ko03050 |
| **47** | Propanoate metabolism | 102 (1.25%) | ko00640 |
| **48** | RNA polymerase | 101 (1.24%) | ko03020 |
| **49** | Glycerolipid metabolism | 101 (1.24%) | ko00561 |
| **50** | Nucleotide excision repair | 98 (1.21%) | ko03420 |
| **51** | ABC transporters | 96 (1.18%) | ko02010 |
| **52** | beta-Alanine metabolism | 95 (1.17%) | ko00410 |
| **53** | Butanoate metabolism | 93 (1.14%) | ko00650 |
| **54** | Porphyrin and chlorophyll metabolism | 92 (1.13%) | ko00860 |
| **55** | Phenylalanine metabolism | 92 (1.13%) | ko00360 |
| **56** | Protein export | 91 (1.12%) | ko03060 |
| **57** | Ubiquinone and other terpenoid-quinone biosynthesis | 88 (1.08%) | ko00130 |
| **58** | Arginine biosynthesis | 86 (1.06%) | ko00220 |
| **59** | N-Glycan biosynthesis | 84 (1.03%) | ko00510 |
| **60** | Fatty acid degradation | 83 (1.02%) | ko00071 |
| **61** | Homologous recombination | 82 (1.01%) | ko03440 |
| **62** | Sulfur metabolism | 81 (1%) | ko00920 |
| **63** | Tyrosine metabolism | 80 (0.98%) | ko00350 |
| **64** | Ascorbate and aldarate metabolism | 78 (0.96%) | ko00053 |
| **65** | alpha-Linolenic acid metabolism | 78 (0.96%) | ko00592 |
| **66** | DNA replication | 78 (0.96%) | ko03030 |
| **67** | Carotenoid biosynthesis | 78 (0.96%) | ko00906 |
| **68** | Mismatch repair | 75 (0.92%) | ko03430 |
| **69** | Fatty acid biosynthesis | 74 (0.91%) | ko00061 |
| **70** | Basal transcription factors | 73 (0.9%) | ko03022 |
| **71** | Cyanoamino acid metabolism | 71 (0.87%) | ko00460 |
| **72** | Regulation of autophagy | 70 (0.86%) | ko04140 |
| **73** | Tryptophan metabolism | 70 (0.86%) | ko00380 |
| **74** | Pantothenate and CoA biosynthesis | 70 (0.86%) | ko00770 |
| **75** | SNARE interactions in vesicular transport | 68 (0.84%) | ko04130 |
| **76** | Base excision repair | 66 (0.81%) | ko03410 |
| **77** | Biosynthesis of unsaturated fatty acids | 63 (0.77%) | ko01040 |
| **78** | Valine, leucine and isoleucine biosynthesis | 61 (0.75%) | ko00290 |
| **79** | Circadian rhythm - plant | 59 (0.73%) | ko04712 |
| **80** | Lysine degradation | 57 (0.7%) | ko00310 |
| **81** | Nitrogen metabolism | 56 (0.69%) | ko00910 |
| **82** | Sphingolipid metabolism | 55 (0.68%) | ko00600 |
| **83** | One carbon pool by folate | 53 (0.65%) | ko00670 |
| **84** | Tropane, piperidine and pyridine alkaloid biosynthesis | 50 (0.61%) | ko00960 |
| **85** | Selenocompound metabolism | 48 (0.59%) | ko00450 |
| **86** | Histidine metabolism | 47 (0.58%) | ko00340 |
| **87** | Other glycan degradation | 46 (0.57%) | ko00511 |
| **88** | Biotin metabolism | 45 (0.55%) | ko00780 |
| **89** | Steroid biosynthesis | 44 (0.54%) | ko00100 |
| **90** | Nicotinate and nicotinamide metabolism | 41 (0.5%) | ko00760 |
| **91** | Fatty acid elongation | 40 (0.49%) | ko00062 |
| **92** | Photosynthesis - antenna proteins | 40 (0.49%) | ko00196 |
| **93** | Ether lipid metabolism | 39 (0.48%) | ko00565 |
| **94** | Isoquinoline alkaloid biosynthesis | 37 (0.45%) | ko00950 |
| **95** | Folate biosynthesis | 36 (0.44%) | ko00790 |
| **96** | Thiamine metabolism | 34 (0.42%) | ko00730 |
| **97** | Glycosylphosphatidylinositol(GPI)-anchor biosynthesis | 34 (0.42%) | ko00563 |
| **98** | Lysine biosynthesis | 34 (0.42%) | ko00300 |
| **99** | Sulfur relay system | 32 (0.39%) | ko04122 |
| **100** | Diterpenoid biosynthesis | 29 (0.36%) | ko00904 |
| **101** | Arachidonic acid metabolism | 29 (0.36%) | ko00590 |
| **102** | C5-Branched dibasic acid metabolism | 28 (0.34%) | ko00660 |
| **103** | Vitamin B6 metabolism | 28 (0.34%) | ko00750 |
| **104** | Cutin, suberine and wax biosynthesis | 27 (0.33%) | ko00073 |
| **105** | Riboflavin metabolism | 26 (0.32%) | ko00740 |
| **106** | Stilbenoid, diarylheptanoid and gingerol biosynthesis | 26 (0.32%) | ko00945 |
| **107** | Flavonoid biosynthesis | 25 (0.31%) | ko00941 |
| **108** | Linoleic acid metabolism | 25 (0.31%) | ko00591 |
| **109** | Monobactam biosynthesis | 24 (0.3%) | ko00261 |
| **110** | Zeatin biosynthesis | 23 (0.28%) | ko00908 |
| **111** | Glycosaminoglycan degradation | 23 (0.28%) | ko00531 |
| **112** | Taurine and hypotaurine metabolism | 21 (0.26%) | ko00430 |
| **113** | Degradation of aromatic compounds | 20 (0.25%) | ko01220 |
| **114** | Sesquiterpenoid and triterpenoid biosynthesis | 17 (0.21%) | ko00909 |
| **115** | Non-homologous end-joining | 16 (0.2%) | ko03450 |
| **116** | Limonene and pinene degradation | 14 (0.17%) | ko00903 |
| **117** | Brassinosteroid biosynthesis | 14 (0.17%) | ko00905 |
| **118** | Glycosphingolipid biosynthesis - globo series | 13 (0.16%) | ko00603 |
| **119** | Synthesis and degradation of ketone bodies | 10 (0.12%) | ko00072 |
| **120** | Lipoic acid metabolism | 9 (0.11%) | ko00785 |
| **121** | Other types of O-glycan biosynthesis | 9 (0.11%) | ko00514 |
| **122** | Caffeine metabolism | 8 (0.1%) | ko00232 |
| **123** | Glycosphingolipid biosynthesis - ganglio series | 6 (0.07%) | ko00604 |
| **124** | Monoterpenoid biosynthesis | 6 (0.07%) | ko00902 |
| **125** | Anthocyanin biosynthesis | 5 (0.06%) | ko00942 |


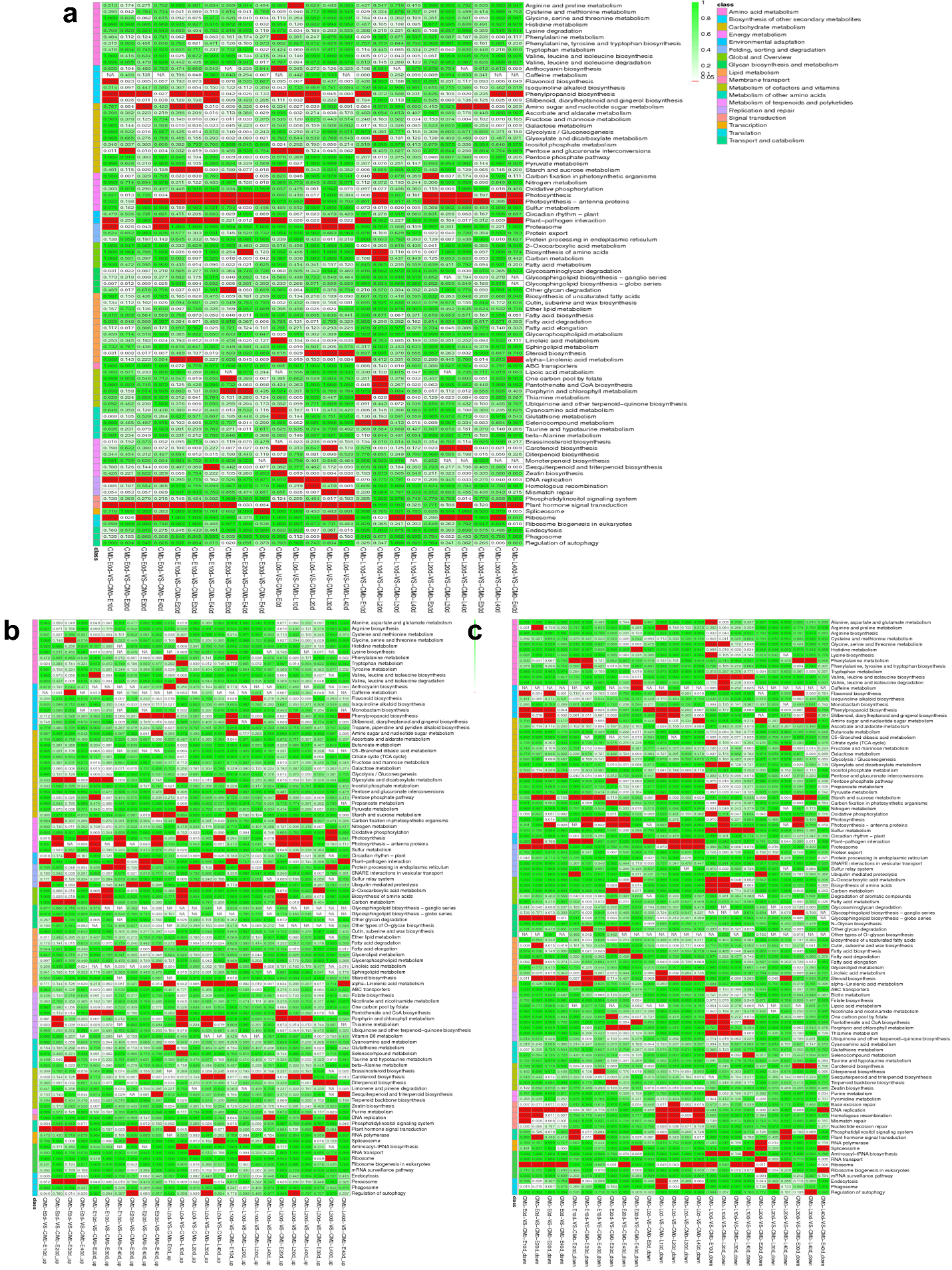


**Fig. S2.** KEGG pathways enrichment analysis of DEGs of *C. moschata*. **(a)** KEGG pathways enrichment of total DEGs in all pairwise comparisons of CMO-X and CMO-E, **(b)** KEGG pathways enrichment of upregulated DEGs in all pairwise comparisons of CMO-X and CMO-E and **(c)** KEGG pathways enrichment of downregulated DEGs in all pairwise comparisons of CMO-X and CMO-E.

**Table S4** Genes involved in sucrose metabolism

| **Gene abbreviation** | **Unigene ID** |
| --- | --- |
| *SUS* | Unigene0000027, Unigene0020193, Unigene0033931, Unigene0035690, Unigene0039025, Unigene0044421, Unigene0040240, Unigene0016856, Unigene0020669, Unigene0036510, Unigene0038288 |
| *SPS* | Unigene0018484, Unigene0020669, Unigene0038286, Unigene0038287, Unigene0038288, Unigene0036510, Unigene0036511, Unigene0040240, Unigene0040241, Unigene0020193 |
| *INV* | Unigene0002448, Unigene0003950, Unigene0004188, Unigene0004189, Unigene0005556 , Unigene0020720, Unigene0030378 , Unigene0030379 , Unigene0044132, Unigene0052914, Unigene0020055 |
| *PGI* | Unigene0010966, Unigene0010967, Unigene0010968, Unigene0012171, Unigene0030474, Unigene0030473, Unigene0030475 |
| *UGPase* | Unigene0000830, Unigene0002219 |
| *PGM* | Unigene0002629, Unigene0037284 |
| *HK* | Unigene0002583, Unigene0032614, Unigene0032613, Unigene0032611, Unigene0028620, Unigene0028619, Unigene0026256, Unigene0026255, Unigene0026254, Unigene0020260, Unigene0006910, Unigene0032612, Unigene0034895, Unigene0003204 |
| *FK* | Unigene0052295, Unigene0046036, Unigene0045940, Unigene0026172, Unigene0026171, Unigene0021526, Unigene0021525, Unigene0013215, Unigene0036385, Unigene0045941, Unigene0050825 |
| *AGPase* | Unigene0047165, Unigene0040458, Unigene0040454, Unigene0039030, Unigene0031387, Unigene0016505, Unigene0003314 |

**
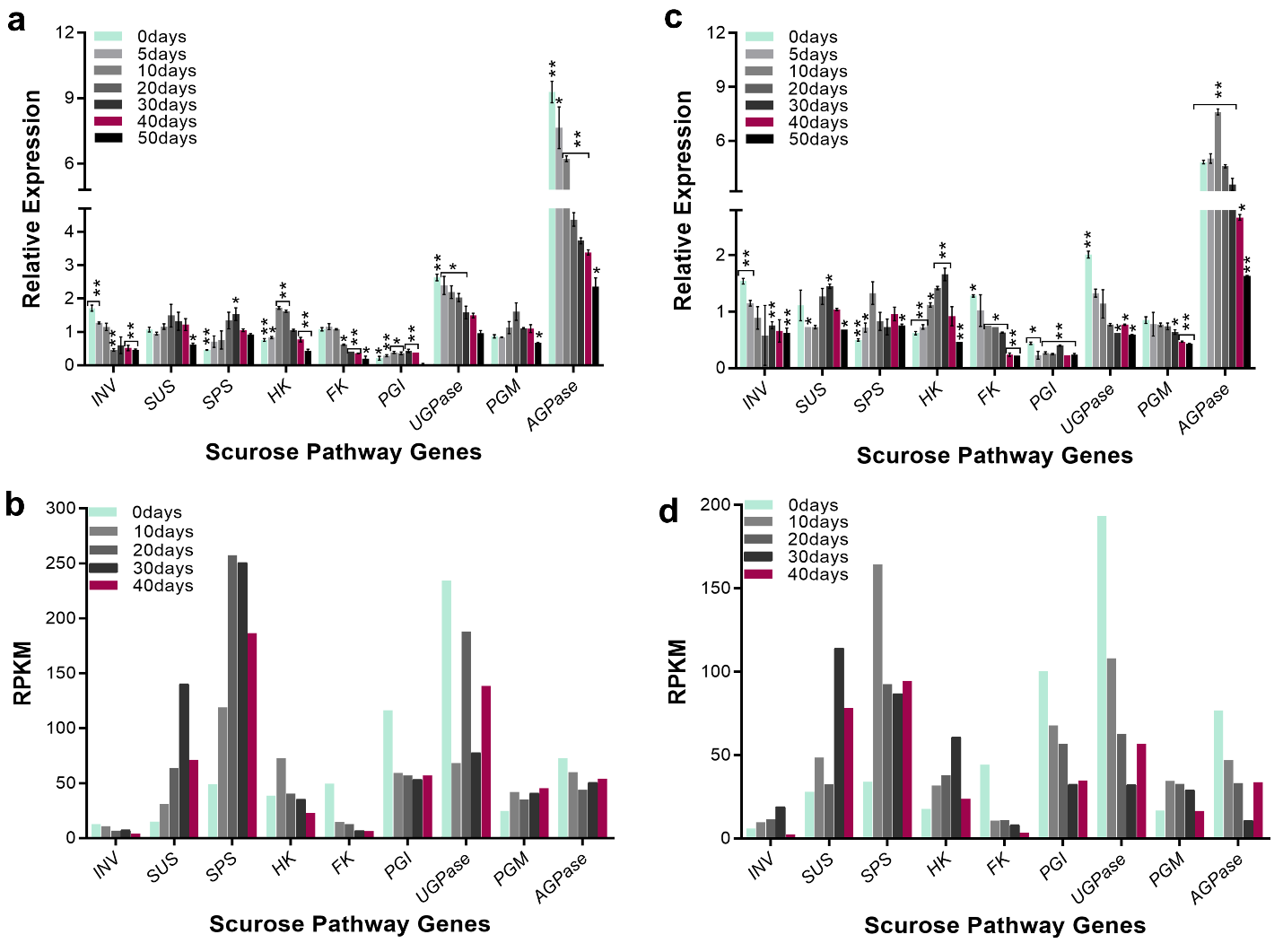
**

**Fig. S3** Relative expression and RPKM of different genes from sucrose pathway.

Samples were collected at different stages of fruit development. RNA was extracted and cDNA was synthesized for qRT-PCR. Actin was used as internal control. **(a)** Relative expression of genes from sucrose pathways in CMO-X, **(b)** RPKM values of genes from sucrose pathways in CMO-X, **(c)** Relative expression of genes from sucrose pathways in CMO-E, and **(d)** RPKM values of genes from sucrose pathways in CMO-E. Significance was determined by *t*-test **P*<0.05, ***P*<0.01. Results are the mean values from three independent experiments. Vertical bars represent SD.

**Table S5** Genes involved in carotenoids biosynthesis pathways

| **Gene abbreviation** | **Unigene ID** |
| --- | --- |
| *PSY* | Unigene0032133, Unigene0032135, Unigene0041547, Unigene0046580, Unigene0028991, Unigene0000357, Unigene0030641 |
| *BOH* | Unigene0025843, Unigene0031912 |
| *EOH* | Unigene0042540, Unigene0055096 |
| *LUT1* | Unigene0025967, Unigene0042540, Unigene0055096, Unigene0031399, Unigene0031400 |
| *LCYE* | Unigene0031051, Unigene0031052 |
| *LCYB* | Unigene0018220, Unigene0018221 |
| *ZDS* | Unigene0035295, Unigene0035296, Unigene0046817, Unigene0035297 |
| *PDS* | Unigene0036921, Unigene0039091, Unigene0000134, Unigene0003036 |
| *ZEP* | Unigene0003183, Unigene0040268, Unigene0040770, Unigene0040771, Unigene0015448, Unigene0016123 |
| *VDE* | Unigene0053139, Unigene0053140 |
| *CRTISO* | Unigene0036596, Unigene0023651, Unigene0034384 |
| *CCD8* | Unigene0050566, Unigene0000595, Unigene0016849, Unigene0020974, Unigene0021011, Unigene0035128, Unigene0037104, Unigene0037105, Unigene0000143, Unigene0045420 |


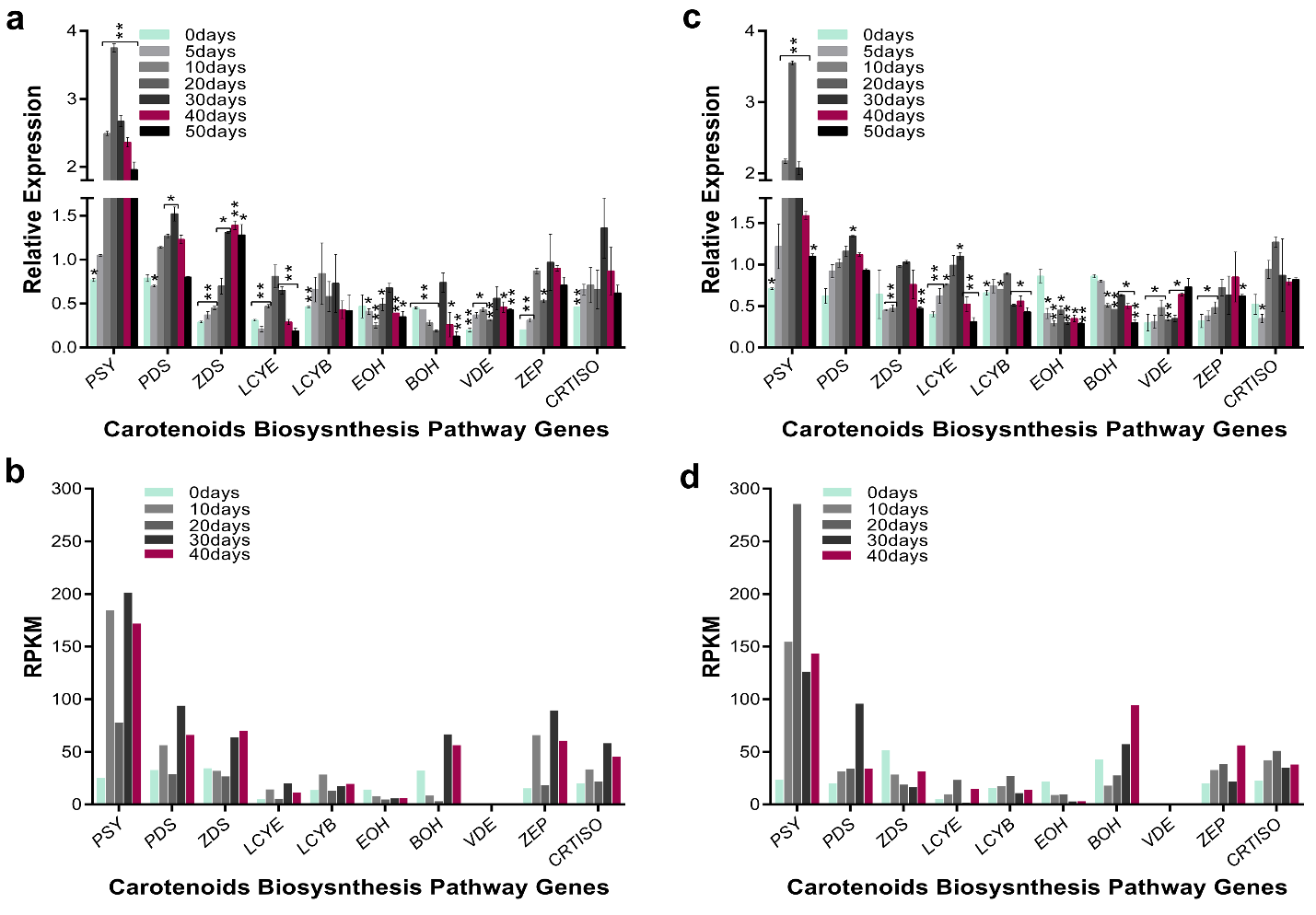


**Fig. S4** Relative expression and RPKM of different genes from carotenoids biosynthesis pathway. Samples were collected at different stages of fruit development. RNA was extracted and cDNA was synthesized for qRT-PCR. Actin was used as internal control. **(a)** Relative expression of genes from carotenoids biosynthesis pathway in CMO-X, **(b)** RPKM values of genes from carotenoids biosynthesis pathway in CMO-X, **(c)** Relative expression of genes from carotenoids biosynthesis pathway in CMO-E, and **(d)** RPKM values of genes from carotenoids biosynthesis pathway in CMO-E. Significance was determined by *t*-test **P*<0.05, ***P*<0.01. Results are the mean values from three independent experiments. Vertical bars represent SD.

**Table S6** List of primers used for qRT-PCR

| **Gene** | **Primer sequence (5’-3’)** |
| --- | --- |
| *Actin-F* | CGGCCATTGAGAAAAGCTACGAACT |
| *Actin-R* | CCCACCACTGAGGACGATGTTACCA |
| *INV-F* | CAAGAAGACGGGCAGCAA |
| *INV-R* | GAGCCAGGTTCGAGAAGG |
| *SUS*-F | GGCAGCAAAGATACGG |
| *SUS*-R | CTCCAGGTGAGACGATGT |
| *SPS*-F | TCAAAACCATCATACAGA |
| *SPS*-R | CGGAGGATAGAGGCAGAG |
| *PGM*-F | GCTGGAGCAACTGTAAGAA |
| *PGM*-R | GGACAAACATAATGCCAAA |
| *PGI*-F | TGAAGCCTCAGACCAAG |
| *PGI*-R | AGCTCCAGCACCTAATT |
| *UGPase*-F | TGGTTGGCTCGGATAATGT |
| *UGPase*-R | TGGAGTTGAATAAGTGCTGGT |
| *AGPase*-F | ATCACTGTAGCTGCATTACCC |
| *AGPase*-R | TCAACCTTCATCGCTTTCA |
| *HK*-F | CGATGTGATTGCTGCTGTG |
| *HK*-R | GAGGACCGGAAGTTACCC |
| *FK*-F | TAGCGTTTGTTACGTTGA |
| *FK*-R | GATCTTTGCCTTCTTGAT |
| *BOH*-F | GATTCTCCTGGCAACTCA |
| *BOH*-R | TCGCCCTATGATGAGACTTG |
| *EOH*-F | GTGTAAGATAATCCCAAGAC |
| *EOH*-R | TTCACATATTCCTCCTCAT |
| *VDE*-F | AAACAGCGTCGTGGATG |
| *VDE*-R | AACAGACGGGTCGGGTA |
| *PSY*-F | CCAAACATTGGACGAGATTG |
| *PSY*-R | GGAGAGACCTTGCATAAGCC |
| *PDS*-F | GTTTAGCAGGAGTCCGCTTC |
| *PDS*-R | AAATCCACTCCTCTGCAGGT |
| *ZDS*-F | CAACGGATGGGTTACAGAGA |
| *ZDS*-R | GCAAAGCATGAGAAATCTGC |
| *LCYB*-F | CAACTGCCTGTTCTTCCTCA |
| *LCYB*-R | CAACAATAGGTGCTGCTGCT |
| *LCYE*-F | GATCCTTGCCAAACACAGAG |
| *LCYE*-R | TTTGGAGCCTCAGACAGAGA |
| *ZEP*-F | ATCTCCTGGTTGGAGCTGAT |
| *ZEP*-R | CTGGTATGAAGTCGGCGATA |
| *CRTISO*-F | AGGAGTGGACCTTCCCAAGGA |
| *CRTISO*-R | ATGCTCAGAAAGATGCTTCCAT |
